# Macrophage caspase-8 inhibition accelerates necrotic core expansion in atheroma plaque in mice

**DOI:** 10.3389/fimmu.2025.1513637

**Published:** 2025-04-08

**Authors:** Thomas Pilot, Stéphanie Solier, Antoine Jalil, Charlène Magnani, Tom Vanden Berghe, Peter Vandenabeele, David Masson, Eric Solary, Charles Thomas

**Affiliations:** ^1^ Université de Bourgogne, Center for Translational and Molecular Medicine (CTM) Unité Mixte de Recherche (UMR) 1231, Dijon, France; ^2^ Institut national de la santé et de la recherche médicale (INSERM), UMR1231, Dijon, France; ^3^ LabEx LipSTIC, Dijon, France; ^4^ INSERM, UMR1287, Gustave Roussy, Villejuif, France; ^5^ Inflammation Research Center (IRC), Vlaams Instituut voor Biotechnologie (VIB), Ghent, Belgium; ^6^ Department of Biomedical Molecular Biology, University of Ghent, Ghent, Belgium; ^7^ Department of Biomedical Sciences, Infla-Med Centre of Excellence, University of Antwerp, Antwerp, Belgium; ^8^ Centre Hospitalier Régional Universitaire (CHRU) Dijon Bourgogne, Laboratory of Clinical Chemistry, Dijon, France; ^9^ Faculté de Médecine, Université Paris-Saclay, Le Kremlin-Bicêtre, France

**Keywords:** atherosclerosis, apoptosis, necroptosis, necrotic core, macrophages, caspase-8

## Abstract

**Background and aims:**

Cell death plays a central role in atheroma plaque progression and aggravation. This study investigates the role of caspase-8 in regulating macrophage cell death modalities, specifically apoptosis and necroptosis, within atheroma plaques.

**Methods:**

Bone marrow from caspase-8-deficient (*Casp8^komac^*) and cohoused wildtype littermates were transplanted in atherosclerosis-prone *Ldlr^-/-^* recipient mice fed with a proatherogenic diet. Aortic plaque development, necrotic core formation, and cell death were analyzed through histological and biochemical assays. *In vitro* investigation of macrophages exposed to atherogenic stimuli assessed the effects of caspase-8 inhibition on apoptotic and necroptotic pathways.

**Results:**

Despite lower plasma cholesterol levels and reduced number of inflammatory monocytes, caspase-8-deficient mice exhibited more pronounced atherosclerotic lesions with enlarged necrotic cores and an increased number of dead cells. *In vitro*, in macrophages exposed to oxidized LDL or oxysterols, the inhibition of caspase-8 revealed a shift from apoptosis to necroptosis as confirmed by increased phosphorylation of MLKL along with decreased cleavage of caspase-3 and -7.

**Discussion and perspectives:**

The study highlights the role of caspase-8 in atherosclerosis in tuning the balance between apoptosis and necroptosis. Caspase-8 inhibition leads to a switch towards necroptosis and accumulation of dead cell corpses that contributes to enhanced plaque severity. These findings suggest that reducing caspase-8-regulated necroptosis and necrosis in macrophages could represent a therapeutic strategy to stabilize plaques and reduce cardiovascular risk.

## Introduction

Macrophages play a critical role in the initiation and progression of atherosclerosis, a leading cause of mortality worldwide (1). Upon encountering oxidized low-density lipoproteins (oxLDLs), macrophages evolved into foam cells within the intimal layer of arteries (2). Accumulation of foam cells is a hallmark of atherosclerotic plaques. As plaques progress, foam cells eventually undergo different forms of cell death that contribute to plaque severity. Efferocytosis, the process by which macrophages clear dead cells, plays a crucial role in preventing or limiting the formation of a necrotic core within the plaque, which is strongly associated with cardiovascular events such as myocardial infarction and stroke. However, when efferocytosis is defective or overwhelmed, the accumulation of dead cells contributes to the expansion of the necrotic core and the plaque instability (3).

Multiple forms of macrophage cell death are implicated in atherosclerosis, with the dominant form varying depending on the stage of the disease. These cell death modalities include apoptosis, ferroptosis, pyroptosis, parthanatos, and necroptosis, as well as secondary necrosis, which results from the failed clearance of dead cells in advanced atheroma lesions (4, 5). The stimuli inducing cell death are not yet fully understood but are closely linked to the plaque microenvironment. Factors such as hypoxia, oxidative stress, and exposure to cytotoxic lipid molecules, including oxidized phospholipids and oxysterols, are known to play significant roles. Apoptosis, the best-characterized form of regulated cell death, is associated with cell shrinkage, chromatin condensation, and membrane blebbing. Apoptosis can be triggered through extrinsic (e.g., death receptor signaling) or intrinsic pathways (e.g., mitochondrial stress). Both converge on the activation of effector caspases, notably caspases-3 and -7 (5). Caspase-8 plays a central role in the initiation of apoptosis by cleaving and activating these downstream effector caspases (4). Importantly, caspase-8 also negatively affects necroptosis, another form of regulated cell death with pro-inflammatory consequences. Necroptosis differs from apoptosis in that it is characterized by the rapid release of damage-associated molecular patterns (DAMPs) and pro-inflammatory cytokines. This inflammatory form of cell death is typically initiated when caspase-8 is inhibited, and it depends on the formation of a protein complex named necrosome. This complex includes receptor-interacting protein kinases RIPK1 and RIPK3, as well as MLKL, which, upon phosphorylation by RIPK3, causes plasma membrane permeabilization, leading to cell death (6–8). Caspase-8 inhibits necroptosis by cleaving RIPK1 and RIPK3, preventing the assembly of the necrosome. In this process, caspase-8 forms a complex with FADD (Fas-associated death domain) and cFLIP (cellular FLICE-inhibitory protein), which blocks RIPK3 activation and thus halts necroptosis induction. As a result, caspase-8 tends to promote apoptosis over necroptosis when both pathways are engaged (9–11). Conversely, caspase-8-dependent inhibition of necroptosis may reduce inflammation by limiting the release of DAMPs and pro-inflammatory cytokines from necrotic cells within atheroma plaques. In agreement with this assumption, it was recently shown that the inhibition of RIPK3 in macrophages leads to a drop in the size of atherosclerotic lesions by diminishing primary necrosis in advanced plaques. Interestingly, RIPK3-dependent macrophage necrosis resulted from a shift from apoptosis to necrosis rather than from post-apoptotic cell death (12). Conversely, the inhibition of MLKL, another key player of necroptosis, was associated with decreased necrotic core formation in advanced atherosclerotic lesions in mice (13). In this study, we aimed to investigate the role of necroptosis in the progression of atherosclerosis by silencing or inhibiting caspase-8 in mice or primary macrophages in order to promote a switch towards necroptosis. Bone marrow transplantation experiments were performed using donor mice displaying a targeted deficiency of *caspase-8* in myeloid cells (*Casp8^komac^*) and athero-prone *Ldlr^-/-^* recipient mice. Our results revealed that *Ldlr^-/-^* mice grafted with *Casp8^komac^* bone marrow exhibited more atherosclerotic lesions with larger necrotic core than mice grafted with wild-type bone marrow from cohoused littermates. In accordance with caspase-8-dependent modulation of cell death, *Ldlr^-/-^* mice grafted with *Casp8^komac^* bone marrow displayed a higher proportion of dead cells. *In vitro*, primary macrophages treated with a specific caspase-8 inhibitor and exposed to oxLDLs or 7-ketocholesterol (a major oxysterol found in atherosclerotic plaques) showed a shift from apoptosis to necroptosis. Our results show the key role of the balance between the different macrophage death modalities and highlight the impact of an unbalance between necroptosis and apoptosis on plaque progression and stability.

## Materials and methods

### Mouse strain and breeding

Caspase-8^flox/flox^ mice were kindly provided by Hedrick’s laboratory (UCSD) (14) and crossed with LysMCre transgenic mice (15) to generate cohoused caspase-8^komac^ littermates (caspase-8^flox/flox-LysM-Cre^), recipient *Ldlr^-/-^* female mice were obtained from The Jackson Laboratory. All mice used were maintained on a C57BL/6J genetic background. Animal procedures were performed in compliance with the ethical guidelines approved by Ethics Committee for the Use of Laboratory Animals of the University of Burgundy (Protocol No. 2017032015521461_v1#8381). Mice were fed either a standard chow diet (A3, Safe) or a Western diet (U8958, Safe) starting at 8 weeks of age.

### Bone marrow transplantation

Eight weeks old *Ldlr^-/-^* female recipient mice were irradiated with a dose of 11 Gy. Subsequently, the mice received a tail vein injection of 2x10^6^ bone marrow cells isolated from *caspase-8^flox/flox^* and *caspase-8^komac^* male mice. A recovery period of 4 weeks followed the injection, during which the drinking water was supplemented with enrofloxacin antibiotic (100 mg/L), renew on a weekly basis.

### Atherosclerosis study

After the recovery period, the mice were fed with a western type diet (U8958, Safe) containing: 7% PM 205B SAFE; 1% PV 200 SAFE; 17% casein acid; 0.3% methionine; 34% saccharose; 14.5% corn starch; 0.2% cholesterol; 5% cellulose; 21% dairy butter for 12 weeks. At the end of the dietary intervention, mice were anesthetized with isoflurane and blood samples were collected via intracardiac puncture. The mice were then euthanized by cervical dislocation and the bones, heart, aorta and carotid arteries were harvested. Heart was perfused with PBS, fixed in 4% paraformaldehyde (PFA embedded in paraffin and sectioned for quantification of aortic valves with hematoxylin-eosin (HE) staining. Quantitative analysis was performed using NDP.View2 software, version 2.7.25 from Hamamatsu by three independent investigators blinded to the experimental conditions. Aorta was fixed in 4% PFA for en face staining.

### Bone-marrow-derived-macrophages preparation

*Casp8^flox/flox^*, *Casp8^komac^* and wild type C57BL/6J mice were anesthetized under isoflurane and euthanized by cervical dislocation. Femurs and tibias were harvested, and bone marrow cells were flushed out using RPMI 1640 medium supplemented with 5% FBS and 1% penicillin-streptomycin. Cells were seeded at a density of 5x10^5^ in 12-well plates or 1x10^6^ cells in 6-well plates. Differentiation into macrophages was induced by adding recombinant M-CSF (130-096-492, Miltenyi) for 5 to 6 days until complete differentiation.

### Plasma lipid analysis

Plasma lipid parameters were assessed using a Victor^2^ 1420 Multilabel Counter (PerkinElmer Life Science, Boston, MA). Cholesterol and triglyceride levels were quantified through enzymatic assays (Diasys; Catalog No. 113009910026, 157109910026), following the manufacturer’s instructions. To analyze lipoprotein fractions, pooled plasma samples from *Casp8^flox/flox^* and *Casp8^komac^* mice were prepared. The separation of lipoproteins was performed using an FPLC system (UPC-900, P-920, Frac-900, AKTA). Cholesterol content in each fraction was subsequently determined using an enzymatic cholesterol assay, as previously described.

### White blood cells count

White blood cell counts were determined from blood samples collected at the time of sacrifice. A 50 µL aliquot was analyzed using a Scil vet abc Plus+ hematology analyzer (Scil).

### Flow cytometry

Erythrocytes were lysed using ACK lysis buffer, and the remaining nucleated cells were harvested. Cells were washed with ice-cold PBS and blocked with Fc receptor blocker (Murine TruStain FcX, Biolegend, diluted 1:50) for 15 minutes. Cells were then incubated with specific antibodies for 20 minutes at 4°C for white blood cell phenotyping. The following antibodies were used: CD45-BUV395 (BD, 564279), CD11b-BV510 (Biolegend, 101245), Ly6G-APC-Cy7 (Biolegend, 127624), Ly6C-PE (Biolegend, 128008), CX3CR1-BV711 (Biolegend, 149031), and CD192-PE-Cy7 (Biolegend, 150612), following the manufacturers’ protocols. After washing, fluorescence was detected using a BD LSR Fortessa X-20, and the data were analyzed using FlowJo software (v10.9.0). The gating strategy is depicted **Supplementary Figure S1**.

### Cytokine measurement

Cytokines were analyzed in the murine plasma, using the Mouse Pro-Inflammatory Panel 1 V-Plex (MSD, Rockville, MD, US). The kit was run according to the manufacturer’s guidelines and the chemi-luminescence signal was measured on a Sector Imager 2400 (MSD).

### Cellular treatment

After 5 to 6 days of differentiation, macrophages derived from C57BL/6J mice were either treated or left untreated with the caspase-8 specific inhibitor Z-IETD-FMK (10 µM; Invivogen) for 2 hours, followed by exposure to 7-ketocholesterol (40 µM; Sigma, 700015P) for either 8 or 18 hours. For *Casp8^flox/flox^* and *Casp8^komac^* mice, macrophages were differentiated and treated with ox-LDL (50 µg/mL) for 18 hours. Cells were then harvested for subsequent western blot or flow cytometry analysis.

### Cellular death assessment

A TUNEL assay was performed on murine valves using the Apoptag Peroxidase *In Situ* Apoptosis Detection Kit (Sigma, S700), following the manufacturer’s protocol. Two independent investigators, blinded to the experimental conditions, quantified cell death. Additionally, apoptosis in bone-marrow-derived macrophages (BMDMs) was assessed using the FITC Annexin V Apoptosis Detection Kit (BD, 556547). After treatment, 5x10^5^ cells were washed with PBS and resuspended in 1X Annexin V binding buffer containing CaCl_2_. Each sample was incubated with 5 µL of FITC Annexin V and 5 µL of Propidium Iodide (PI) for 15 minutes at room temperature, in the dark. Finally, 400 µL of 1X binding buffer was added, and fluorescence was measured using a BD LSR Fortessa. Data were analyzed with FlowJo software (v10.9.0).

### Western blot analysis

Following treatment, 1x10^6^ cells were harvested in 100 µL of RIPA buffer (Fisher, 10230544) supplemented with a protease inhibitor (Fisher, 15634189) and Halt phosphatase inhibitor cocktail (Fisher, 10668304). After centrifugation at 15,000 g for 10 minutes, protein quantification was performed using a BCA assay. A total of 10 to 20µg of protein was mixed with loading buffer and reducing agent (Invitrogen, 2550989-2550991). SDS-PAGE was conducted, and proteins were transferred onto a 0.2 µm PVDF membrane (Bio-Rad, 1704272) using a Bio-Rad transblot system. Membranes were blocked with PBS containing 5% BSA and 0.1% Tween-20 for 1 hour at room temperature, followed by incubation overnight at 4°C with primary antibodies (diluted 1:1000 in PBS-5% BSA-0.1% Tween-20). After washing with PBS-0.1% Tween-20, membranes were incubated with HRP-conjugated secondary antibodies (1:2000) for 2 hours. Detection was performed using Pierce ECL substrate (Fisher, 10005943) or SuperSignal West Atto Ultimate Sensitivity Substrate (Fisher, 171181819). The following primary antibodies were used: anti-caspase-3 (Cell Signaling, 9661), anti-caspase-7 (Cell Signaling, 9492), anti-caspase-8 (Cell Signaling, 4790), anti-RIP (Cell Signaling, 3493), anti-RIP3 (Abcam, ab62344), anti-phospho-MLKL (Abcam, ab196436), anti-MLKL (Abcam, ab184718), anti-caspase-1 (Abcam, ab179515), anti-GSDMD (Novus, NBP2-33422) and anti-actin (Abcam, ab49900). HRP-conjugated secondary antibodies included anti-goat (Abcam, ab97100) and anti-rabbit (Cell Signaling, 7074). Caspase 7 and 8 were sequentially revealed from the same membranes after an intermediate stripping step. The same process was applied for pMLKL and MLKL. Non-specific bands are marked with an asterisk. For RIPK1 and RIPK3, the membranes were sectioned at the appropriate molecular weights before hybridization and detection. Uncropped images of the membranes are provided in **Supplementary Figure S2**.

### Immunohistochemistry

Serial sections of mouse heart tissue were prepared and subjected to immunohistochemical staining to evaluate MLKL and phosphorylated-MLKL (pMLKL) expression. Tissue sections were incubated with primary antibodies 1 hour at 1/200 in TBST-BSA 1%: anti-p-MLKL (NBP2-66953, Novus Biologicals) and anti-MLKL (3D4C6, Proteintech). After washing, sections were incubated with ImmPRESS^®^ polymer reagents for IHC (Vector Labs, MP-7401, MP-2400) followed by detection using Vector NovaRED^®^ substrate kit, peroxidase HRP (Vector Labs, SK-4800) as a chromogen. Sections were then counterstained with hematoxylin, dehydrated and mounted. Stained slides were scanned using a high-resolution digital slide scanner. Image analysis was performed using Aperio ImageScope software (v12.4.6.5003). Unsupervised quantification of chromogen-positive staining and total cell count within atheroma plaque was performed using ImageJ software (v1.53V with Java 1.8.0_322).

### Statistical analysis

Data from one representative experiment from 2 to 7 independent experiments were analyzed using GraphPad Prism 10. The number of biological replicates is described in the corresponding figure legend. Results are presented as mean ± SEM. Normality of data distribution was assessed using the Shapiro-Wilk test. If the data followed a normal distribution, statistical comparisons were performed using unpaired or paired t-test as appropriate. If normality was not met, Mann-Whitney U tests (for unpaired comparisons) or Wilcoxon signed-rank tests (for paired comparisons) were applied. A p-value < 0.05 was considered statistically significant.

## Results

### Caspase-8 invalidation in myeloid cells increases cell death and necrotic core formation in atheroma plaques

**Figure 1:**
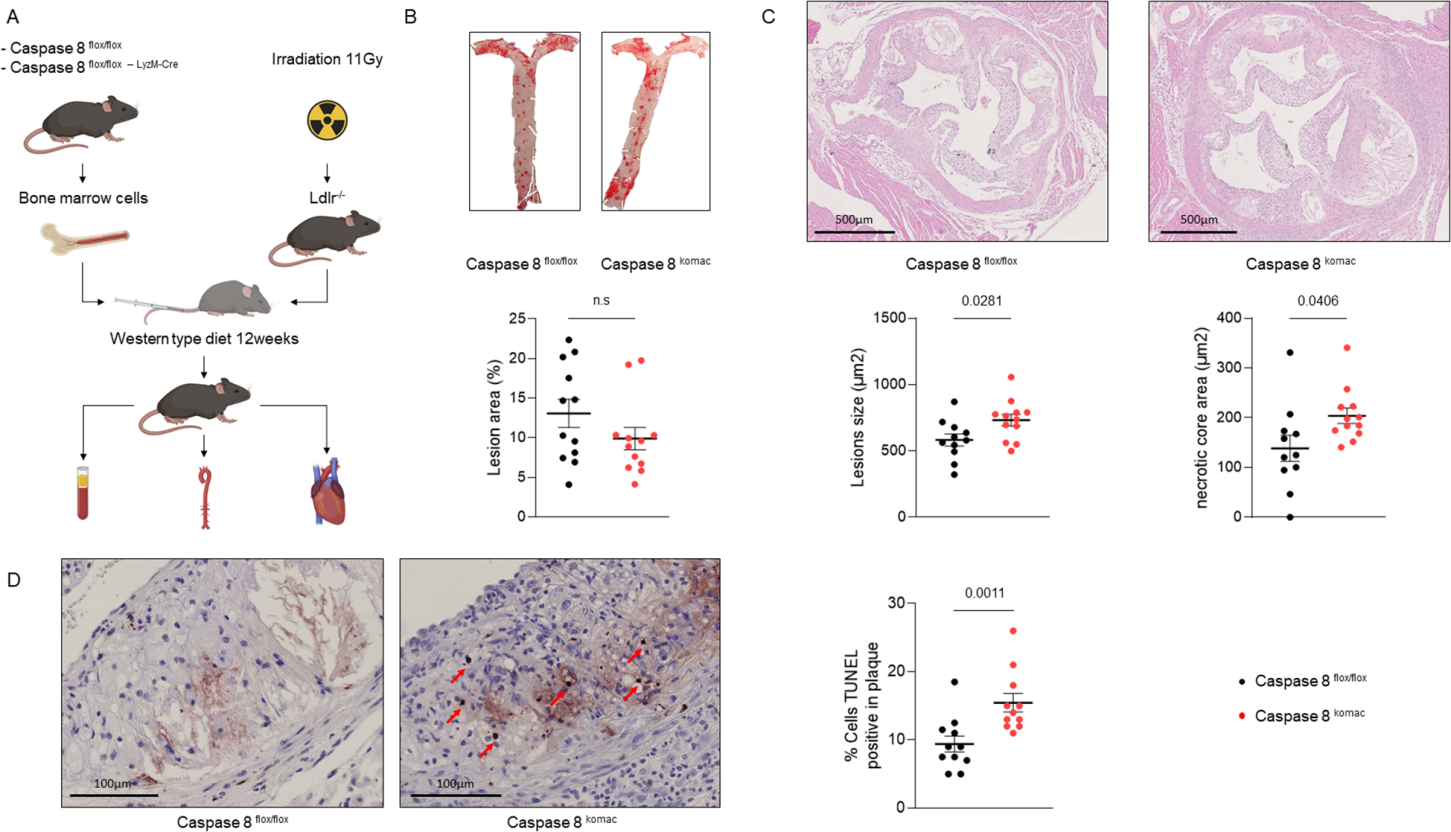
Caspase-8 invalidation in myeloid cells increases necrotic core formation and TUNEL positive cells in atheroma plaques. **(A)**. Experimental strategy. **(B)**. En face staining quantification of the aortas of *Ldlr^-/-^* mice transplanted with *Casp8^flox/flox^* or *Casp8^komac^* hematopoietic cells fed an atherogenic diet for 12 weeks (n=12 per group). **(C)**. Quantification of atheromatous lesion size and necrotic core area in the aortic valves from mice belonging to the same experimental groups than in **(B)** Upper panel, representative images of hematoxylin/eosin-stained aortic valves, scale bar = 500µm. Lower panel, quantification of lesion size, necrotic core area on aortic valves of mice as described in B (*Casp8^flox/flox^* n=11; *Casp8^komac^* n=12). **(D)**. Quantification of cell death by TUNEL assay within atheroma plaque of mice as described in B, scale bar = 100 µm (n=11). Graphs show individual values plus mean ± SEM. Statistical significance was analyzed with Mann-Withney U test when the normality assumption was not met, whereas an unpaired Student’s t-test was applied when normality was achieved. p values between experimental groups are depicted above graphs. ns, non-significant.

*Ldlr^-/-^* mice were transplanted with bone marrow from either *Casp8^komac^* or *Casp8^flox/flox^* cohoused littermate donor mice and subjected to a western-type diet for 12 weeks. Following the dietary period, mice were sacrificed to assess the progression of atherosclerotic lesions (**Figure 1A**). Quantification of lesions in the aortas revealed no significant differences of their number and extent between two groups (**Figure 1B**). However, aortic valve plaques demonstrated a marked increase in lesion size in *Casp8^komac^* mice as compared to control littermates. The absolute necrotic core area was also significantly larger in the *Casp8^komac^* group (**Figure 1C**). To further evaluate whether this increased size of the necrotic core within atheroma plaque could be explained by enhanced necrotic cell death following deficiency of caspase-8, TUNEL assays were performed on the aortic valves. *Casp8^komac^* mice exhibited an increased number of TUNEL-positive cells compared to controls (**Figure 1D**). Noteworthy, while TUNEL staining is typically used to detect apoptotic cells, it also identifies necroptotic and necrotic cells, given that DNA fragmentation occurs in these cell death pathways as well (16, 17). Collectively, these results suggest that ablation of caspase-8 promotes increased cell death, leading to greater necrotic core formation and exacerbated necrotic damage within atheroma plaques.

### Ablation and inhibition of caspase-8 shifts macrophage cell death from apoptosis to necroptosis and necrosis in response to atherogenic stimuli.

**Figure 2:**
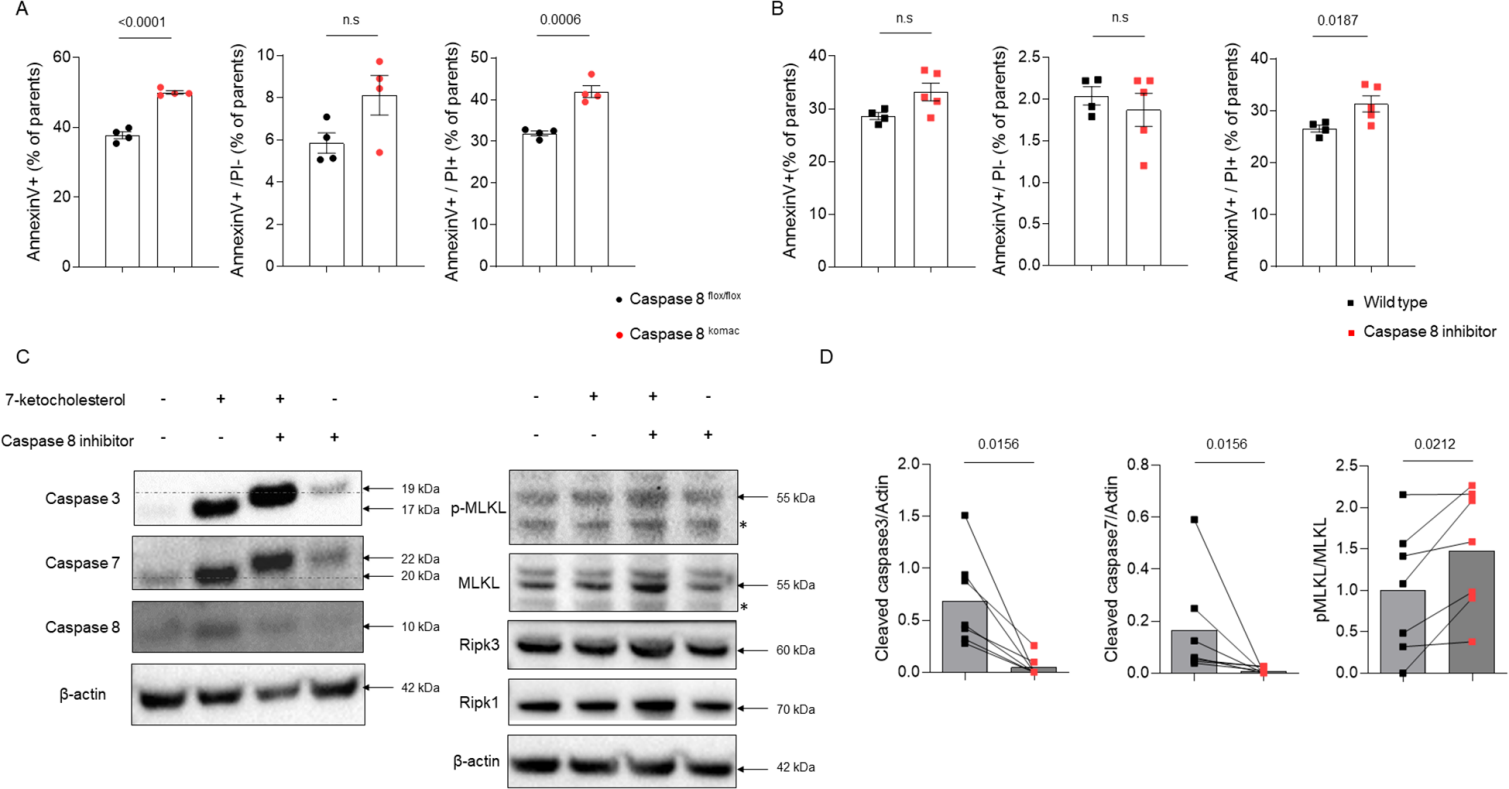
Inhibition of caspase-8 shifts macrophage cell death from apoptosis to necroptosis in response to atherogenic stimuli. **(A)**. Characterization of cell death by flow cytometry using Annexin V/PI staining in bone-marrow-derived macrophages (BMDMs) generated from *Casp8^flox/flox^* or *Casp8^komac^* mice (n=4 independent animal per group). Macrophages were differentiated for 6 days and then exposed to vehicle or ox-LDL at 50µg/mL for 18 hours. Bar graphs show individual values plus mean ± SEM. Statistical significance was analyzed with Mann-Withney U test when the normality assumption was not met, whereas an unpaired Student’s t-test was applied when normality was achieved. **(B)**. Characterization of cell death in BMDMs from C57/BL6J mice (n=5) exposed to 7-ketocholesterol at 40µM for 18 hours in combination or not with the caspase-8 inhibitor Z-IETD-FMK at 10µM for 2 hours before. Bar graphs show individual values plus mean ± SEM. Statistical significance was analyzed with Mann-Withney U test when the normality assumption was not met, whereas an unpaired Student’s t-test was applied when normality was achieved. **(C)**. Western blot analysis of apoptotic and necroptotic checkpoints in BMDMs from C57/BL6J mice exposed to 7-ketocholesterol at 40µM in combination or not with Z-IETD-FMK, 8 hours for necroptotic checkpoints or 18 hours for apoptotic checkpoints. ß-actin was used for normalization of protein levels. Data are representative of 5 independent experiments. Non-specific bands are marked with an asterisk. **(D)**. Western blot quantification of caspase-3 and caspase-7 cleaved forms normalized to ß-actin and MLKL phosphorylation under the same conditions as described in **(C)** Quantification was performed using Image Lab. (caspase-3, caspase-7, MLKL and pMLKL n=7). Bar graphs show individual values plus mean ± SEM. Statistical significance was analyzed with Wilcoxon test when normality assumption was not met, whereas a paired Student’s t-test was applied when normality was achieved. p values between experimental groups are depicted above graphs.

To explore how caspase-8 deficiency influences macrophage cell death pathways, bone marrow-derived macrophages (BMDMs) were generated from *Casp8^komac^* and *Casp8^flox/flox^* mice and exposed to oxLDL to simulate an atherosclerotic environment. After 18 hours of stimulation with 50 µg/mL oxLDL, flow cytometry revealed no significant differences in apoptotic cell death (Annexin V^+^/PI^-^) between the two groups. However, *Casp8^komac^* BMDMs showed a marked increase in necrotic cell populations (Annexin V^+^/PI^+^) compared to *Casp8^flox/flox^* BMDMs (**Figure 2A**). These findings were corroborated by similar experiments using 7-ketocholesterol, a major oxysterol found in atherosclerotic plaques. To mitigate potential compensatory mechanisms arising from genetic knockout, we employed the caspase-8- inhibitor Z-IETD-FMK (10 µM, administered 2 hours before oxysterol exposure), which yielded comparable results. Despite no significant change in apoptosis, necrosis was substantially increased in BMDMs treated with both the caspase-8 inhibitor and 7-ketocholesterol compared to those exposed to 7-ketocholesterol alone (**Figure 2B**). Western blot analyses were performed to validate these observations by assessing key markers of apoptotic and necroptotic pathways. Exposure to 7-ketocholesterol activated apoptotic signaling, as indicated by increased levels of cleaved caspase-3, caspase-7, and caspase-8 in BMDMs (17). However, no significant changes in necroptotic markers were observed at earlier time points, consistent with prior reports suggesting that MLKL phosphorylation precedes necroptosis by several hours (18). Notably, combined treatment with the caspase-8 inhibitor and 7-ketocholesterol resulted in a reduction of apoptotis pathway, as evidenced by inhibited cleavage of caspase-3 and caspase-7. By contrast, the levels of phosphorylated MLKL (pMLKL) and the pMLKL/MLKL ratio were elevated, indicating enhanced necroptotic signaling (**Figures 2C, D**). By contrast, *in vivo*, whereas TUNEL assay revealed an increase in necrotic death in atheroma plaques from *Ldlr^-/-^* mice transplanted with *Casp8^komac^* or *Casp8^flox/flox^* bone marrow (**Figure 1D**), MLKL and pMLKL staining did not show any significant difference in pMLKL/MLKL ratio (**Supplementary Figure S3**). Thus, the early shift from apoptosis to necroptosis caused by caspase 8 inactivation within the plaque microenvironment may ultimately lead to accumulation of dead cell corpses at advanced stages of atheroma development.

Additionally, caspase-8 is known to regulate pyroptosis (19, 20). This pathway depends on caspase-1-dependent cleavage of gasdermin-D. Exposure of BMDMs to 7-ketocholesterol was associated to an increase in the level of the active cleaved form of caspase-1, yet no change was observed in gasdermin-D cleavage (**Supplementary Figure S4**). Furthermore, pharmacological inhibition of caspase-8 in the presence of 7-ketocholesterol did not enhance caspase-1 cleavage but rather led to its reduction (**Supplementary Figure S4**). These findings do not support the occurrence of pyroptotic cell death when caspase-8 is inhibited in macrophages exposed to 7-ketocholesterol or other components of the plaque microenvironment.

In summary, both genetic deletion and pharmacological inhibition of caspase-8 in macrophages shift the balance of cell death regulation from apoptosis towards necroptosis and accumulation of dead cell corpses under atherogenic conditions.

### Caspase-8 invalidation in myeloid cells reduce plasma cholesterol and alters monocyte subsets in mice on an atherogenic diet

**Figure 3:**
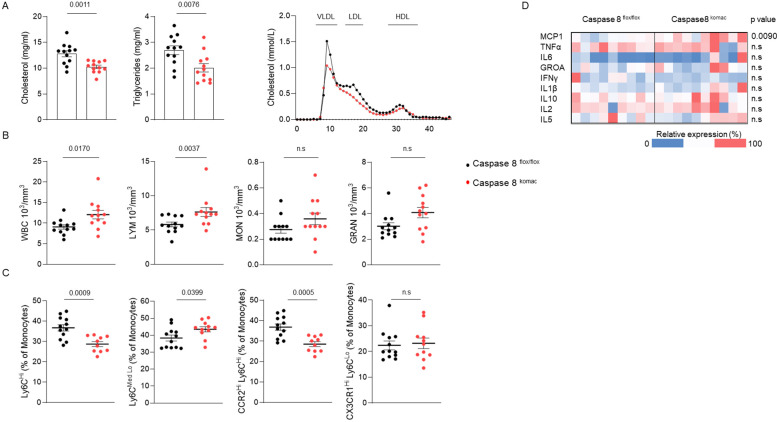
Caspase-8 invalidation in myeloid cells reduces plasma cholesterol and alters monocyte subsets in mice on an atherogenic diet. **(A)**. Plasma lipid composition. **(B)**. blood cell counts of *Ldlr^-/-^* mice transplanted with *Casp8^flox/flox^* or *Casp8^komac^* bone marrow fed an atherogenic diet for 12 weeks (n=12). **(C)**. Immunophenotyping of blood monocytes by flow cytometry from mice as in **(A, B)**. (*Casp8^flox/flox^* n=12; *Casp8^komac^* n=10). **(D)**. Heatmap of plasma cytokine levels of mice as in **(A, B)** (*Casp8^flox/flox^* n=9; *Casp8^komac^* n=10). Bar graphs show individual values plus mean ± SEM. Statistical significance was analyzed with Mann-Whitney U test when the normality assumption was not met, whereas an unpaired Student’s t-test was applied when normality was achieved. p values between experimental groups are depicted above graphs.

Since disturbances of plasma lipid and myeloid cell profiles play a central role in the onset of atherosclerosis, these parameters were investigated in *Casp8^komac^* and *Casp8^flox/flox^* mice fed with an atherogenic diet for 12 weeks. Mice grafted with *Casp8^komac^* bone marrow exhibited lower levels of total cholesterol and triglycerides compared to mice transplanted with bone marrow from *Casp8^flox/flox^* controls (**Figure 3A**). Gel exclusion chromatography further confirmed a significant reduction in cholesterol levels within the VLDL and LDL fractions (**Figure 3A**). Therefore, the atherogenic phenotype associated with *Casp8^komac^* bone marrow engraftment (**Figures 1B, C**) cannot be explained by the plasma lipid profile. Blood cell counts were also performed on these mice. Caspase-8 ablation in myeloid cells is associated with a rise in total white blood cells, with an increased count of lymphocytes whereas the number of monocytes and granulocytes were not significantly altered (**Figure 3B**). Flow cytometry analysis of blood monocytes revealed that myeloid caspase-8 deficiency led to a reduced proportion of inflammatory Ly6C^Hi^ monocytes, while the percentage of patrolling Ly6C^Med/Lo^ monocytes was increased. The decrease in inflammatory monocytes was confirmed by the reduced proportion of CCR2^Hi^Ly6C^Hi^ monocytes, alongside an unchanged percentage of non-classical CX3CR1^Hi^Ly6C^Lo^ monocytes (**Figure 3C**). Overall, phenotypic analysis of blood monocytes from *Casp8^komac^* mice compared to controls showed an increase in patrolling monocytes and a reduction in inflammatory monocytes. The lack of an inflammatory profile in mice transplanted with *Casp8^komac^* bone marrow was further supported by plasma cytokine measurements, which revealed no elevation of inflammatory cytokines typically linked to atherogenesis, as compared to control grafted mice (**Figure 3D**).These findings further confirm that the enhanced atheroma plaque development observed in *Casp8^komac^* bone marrow-grafted *Ldlr^-/-^* mice is strictly due to increased macrophage cell death and related necrotic formation, rather than an aggravated inflammatory profile. Only an elevation in plasma MCP-1 levels was observed, which may contribute to the recruitment of monocytes into atheromatous lesions.

## Discussion

Our study provides novel insights into the role of caspase-8 in regulating cell death pathways within atherosclerotic plaques. To our knowledge, no prior investigation has specifically addressed the impact of myeloid cell-specific caspase-8 ablation in the context of atherosclerosis. Here, we demonstrate that the genetic deletion of caspase-8 in myeloid cells of atherogenic mice promotes a shift from apoptosis to necroptosis and accumulation of dead cell corpses, ultimately contributing to increased necrotic core formation and plaque instability. These findings support the notion that modulating cell death pathways through caspase-8 activity may influence atherosclerosis progression by altering the balance of cell death modalities within the plaque.

Caspase-8 plays a dual role in promoting apoptosis while simultaneously suppressing necroptosis. In our *in vitro* experiments, we observed that caspase-8 deficiency or pharmacological inhibition in macrophages exposed to oxLDL or oxysterols led to a reduction in apoptotic signaling, as evidenced by decreased cleavage of caspase-3 and caspase-7. Concomitantly, we detected an upregulation of MLKL phosphorylation, a hallmark of necroptosis, indicative of a shift towards this cell death pathway. However, despite the increased necrotic cell death (TUNEL-positive cells) observed *in vivo* within atheromatous plaques of *Ldlr^-/-^* mice transplanted with *Casp8^komac^* bone marrow, MLKL and pMLKL staining did not reveal significant differences in the pMLKL/MLKL ratio (**Supplementary Figure S3**).

Several explanations could account for this apparent discrepancy. One possibility is the temporal dynamics of necroptosis, as MLKL phosphorylation occurs at an early stage of the process (18, 21, 22), whereas TUNEL-positive cells and necrotic core formation become apparent at later time points. Consequently, our immunostaining experiments may have failed to capture the peak of MLKL phosphorylation. Moreover, different forms of programmed cell death, including apoptosis, pyroptosis, and necrosis, also result in DNA fragmentation (16, 23), making it challenging to distinguish between these pathways solely based on TUNEL staining. Another potential explanation lies in the recent discovery that ubiquitylated MLKL undergoes proteasome- and lysosome-mediated degradation (24), which could account for the absence of increased pMLKL levels despite a higher rate of cell death. This degradation process might obscure the detection of necroptotic events at later stages, leading to an underestimation of the contribution of necroptosis to plaque pathology.

Furthermore, the accumulation of necrotic debris in *Ldlr^-/-^* mice transplanted with *Casp8^komac^* bone marrow suggests a deficiency in the clearance of dead cells, potentially resulting from impaired efferocytosis or persistent inflammation within the plaque microenvironment. This defect could further contribute to the expansion of the necrotic core and subsequent plaque destabilization. While our *in vitro* findings indicate a shift from apoptosis to necroptosis in macrophages lacking caspase-8, the absence of MLKL phosphorylation *in vivo* raises the possibility that increased cell death within plaques is predominantly driven by extensive apoptosis that progresses to accumulation of dead cell corpses due to defective clearance. Future studies assessing the phagocytic capacity and inflammatory cytokine release in *Casp8^komac^* macrophages will be critical in elucidating the contribution of impaired efferocytosis to plaque necrosis.

**Figure 4:**
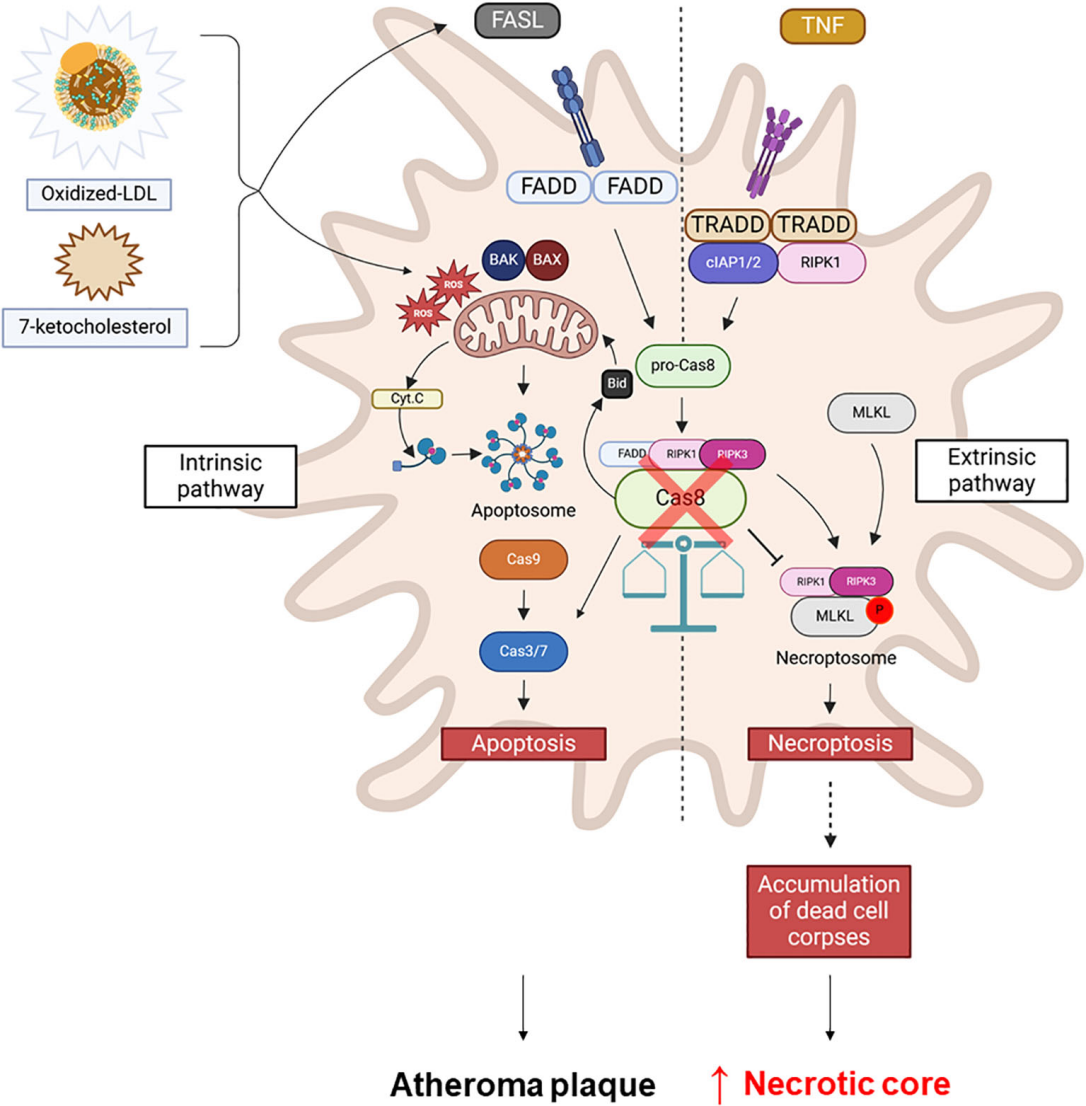
Caspase-8 as a central regulator of macrophage cell death pathways in atherosclerosis. Oxidized LDL (oxLDL) or 7-ketocholesterol induces apoptosis in macrophages, potentially involving the mitochondrial pathway. Caspase-8 plays a key role in this process by regulating the balance between apoptosis and necroptosis by acting as an inhibitor of necroptosis. The inhibition of caspase-8 (red cross) in macrophages in atheroma plaques shifts the cell death program towards necroptosis, leading to necrotic core formation and contributing to plaque instability.

These findings align with both genetic and pharmacological inhibition of caspase-8 activity, highlighting its crucial role in determining macrophage fate within a plaque-like microenvironment (**Figure 4**).

Remarkably, despite the accelerated development of atherosclerotic lesions and the growth of necrotic cores within plaques of mice grafted with *Casp8^komac^* bone marrow, these mice exhibited a generally more favorable lipid and monocyte phenotype. This included lower dyslipidemia and a shift toward a less inflammatory monocyte profile. While the lipid profile of *Casp8^komac^* mice was more favorable with respect to atherosclerosis, this aligns with recent findings that caspase-8 deficiency in myeloid cells, coupled with *Ripk3* knockout, results in lower plasma cholesterol levels (25). Caspase-8 invalidation is known to disrupt immune cell development by affecting hematopoiesis. The connection between plasma cholesterol levels and hematopoiesis, particularly the reduction of plasma cholesterol subsequent to the rise in granulocyte-macrophage progenitors (GMPs), is well-established in atherosclerosis models and may partly explain the phenotype observed in our *Casp8^komac^* group (26–28). In addition, we observed a reduction in Ly6C^Hi^ monocytes in *Casp8^komac^* mice. These inflammatory monocytes are typically recruited to atherosclerotic plaques, where they differentiate into inflammatory macrophages and contribute to plaque progression and instability. The decreased expression of CCR2 in Ly6C^Hi^ monocytes, as observed in our *Casp8^komac^* group, was therefore expected to result in slower plaque progression and smaller lesion sizes. Thus, this observation further underscores the significance of macrophage cell death in atherogenesis.

Our findings are consistent with previous studies that have highlighted the role of necroptosis and the interplay between necroptosis and apoptosis in modulating plaque instability. For instance, inhibition of RIPK3, a key mediator of necroptosis, has been shown to reduce necrotic core formation in advanced plaques. Similarly, MLKL deletion reduces necrotic core areas in advanced lesions (18, 19). Our observation that caspase-8 deficiency leads to larger necrotic cores and increased cell death within plaques suggests that necroptosis or necrosis, rather than being merely a consequence of failed apoptosis, plays an active role in driving plaque progression and instability. Caspase-8 is also implicated in pyroptotic pathway (19, 20). We did not observe any increase in active caspase-1 and cleaved gasdermin-D levels in our model with inhibition of caspase-8 *in vitro* (**Supplementary Figure S4**). These data do not support a role of this cell death modality in the observed phenotype.

The implications of our findings could be of therapeutic significance. The shift from apoptosis to necroptosis likely exacerbates plaque severity by promoting the release of pro-inflammatory cytokines, inflammatory lipid mediators, and damage-associated molecular patterns (DAMPs) from necrotic cells. Thus, targeting necroptosis, either through direct inhibition of MLKL or upstream regulators like RIPK3, could represent a therapeutic strategy to explore in order to stabilize plaques and prevent adverse cardiovascular outcomes.

Several limitations of our study should be noted. While murine models offer mechanistic insights, they may not fully capture human atherosclerosis. Further *in vitro* investigation on the regulation of caspase-8 in pyroptosis, necrosis or its impact on efferocytosis could offer mechanistic insights to better understand the complexity of macrophage death in the context of atheroma plaque. Notably, assessing macrophage associated and free TUNEL-positive cells could help for evaluate the direct effect of caspase-8 deficiency on efferocytosis. Given the critical role of Ninjurin-1 (NINJ1) in plasma membrane rupture, its involvement in macrophage cell death during atherosclerosis deserves further investigation, as impaired clearance of dead cell corpses contributes to disease progression. NINJ1 has been implicated in plasma membrane permeabilization during regulated necrosis and is known to promote membrane rupture and the release of DAMPs during ferroptosis (29, 30). In this context, elucidating the role of NINJ1 in inflammation and plaque progression could provide valuable insights into the development of atherosclerosis. However, markers of necroptosis, such as RIPK1, RIPK3, and MLKL, were reported to be elevated in advanced human plaques. Necroptosis is also active in unstable plaques, and high caspase-8 levels were linked to increased coronary events (31, 32). Investigating the signaling networks and the kinetics between apoptosis and necroptosis is key, as understanding these pathways in chronic inflammation could reveal new therapeutic targets. Translational studies are needed to explore whether modulating these pathways benefits patients with high-risk plaques. In conclusion, our study underscores the critical role of caspase-8 in maintaining the balance between apoptosis and necroptosis within atherosclerotic plaques. Targeting necroptosis may represent a strategy to stabilize plaques and reduce atherosclerosis. As we further understand cell death modalities in plaques, including newer forms like ferroptosis, pyroptosis, or parthanatos, our findings could inspire novel therapies to lower cardiovascular risk.

## Data Availability

The raw data supporting the conclusions of this article will be made available by the corresponding author upon request, without undue reservation.

## Supplementary material

Supplementary Figure 1 Gating strategy and representative flow cytometry from blood monocytes subsets of *Casp8^flox/flox^* and *Casp8^komac^* mice. Gating strategy and representative plots for the identification of blood monocyte subsets. Debris were excluded on the FSC-A and SSC-A parameters (P1), followed by an assessment of acquisition stability using the Time and SSC-A parameters. CD45-positive cells were then gated within the Time, SSC-A subset. Ly6G-negative, CD11b-positive cells were selected from the CD45-positive cells population, corresponding to the monocyte population. Monocyte subsets were then identified based on Ly6C expression (Hi, Med, Low) within the Ly6G-negative, CD11b-positive population. CCR2-positives cells were gated within Ly6C^Hi^, CD11b-positive population and CX3CR1-positives cells on the Ly6C^Lo^, CD11b-positive population.

Supplementary Figure 2 Uncropped images of western blot experiments. Full-length western blot images for the detection of caspase-3, caspase-7, caspase-8, caspase-1, pMLKL, MLKL, RIPK1, RIPK3, GSDMD and corresponding β-actin as presented in Figure3 and FigureS4. The represented lanes 1 to 4 are: 1: control, 2: 7-ketocholesterol, 3: 7-ketocholesterol + Z-IETD-FMK, 4: Z-IETD-FMK. Non-specific bands are marked with an asterisk.

Supplementary Figure 3 Caspase-8 invalidation in myeloid cells impact the expression of pMLKL within atheroma plaque. **(A)**. Ratio of pMLKL-positive cells to MLKL-positive cells in *Casp8^komac^* and *Casp8^flox/flox^* mice (n=12). **(B)**. Representative images of pMLKL and MLKL staining in the valve of *Casp8^komac^* and *Casp8^flox/flox^* mice. MLKL and pMLKL positive cells are stained in red by the chromogen, while cell nuclei are counterstained in blue with hematoxylin. Statistical significance was analyzed with an unpaired-t-test. p values between experimental groups are depicted above the graphs.

Supplementary Figure 4 Inhibition of caspase-8 in primary macrophages impact caspase-1 but not gasdermin-D cleavage. Representative western blot analysis of pro-caspase-1, cleaved fragment p12 and p10 and gasdermin-D, full length (FL) and N-term fragment (NT), in BMDMs from C57/BL6J mice exposed to 40µM 7-ketocholesterol, with or without Z-IETD-FMK, for 18 hours. Nonspecific bands are marked with an asterisk. β-actin was used for protein normalization.
