# Supplementary figures and images for: Macrophage caspase-8 inhibition accelerates necrotic core expansion in atheroma plaque in mice
Source: Front Immunol. 2025 Apr 8;16:1513637. doi: 10.3389/fimmu.2025.1513637 (PMC12011591; doi:10.3389/fimmu.2025.1513637)

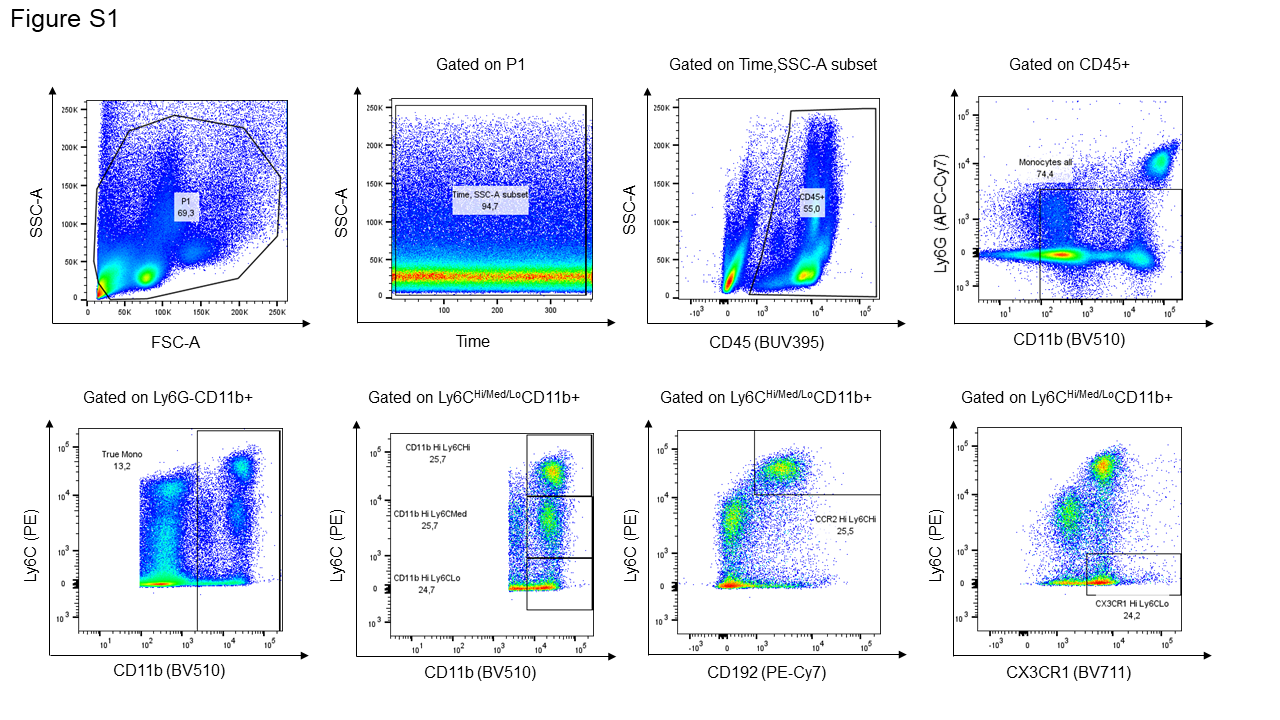

Supplement: Supplementary Figure 1 — Gating strategy and representative flow cytometry from blood monocytes subsets of Casp8flox/flox and Casp8komac mice. Gating strategy and representative plots for the identification of blood monocyte subsets. Debris were excluded on the FSC-A and SSC-A parameters (P1), followed by an assessment of acquisition stability using the Time and SSC-A parameters. CD45-positive cells were then gated within the Time, SSC-A subset. Ly6G-negative, CD11b-positive cells were selected from the CD45-positive cells population, corresponding to the monocyte population. Monocyte subsets were then identified based on Ly6C expression (Hi, Med, Low) within the Ly6G-negative, CD11b-positive population. CCR2-positives cells were gated within Ly6CHi, CD11b-positive population and CX3CR1-positives cells on the Ly6CLo, CD11b-positive population. [file Image1.tif]

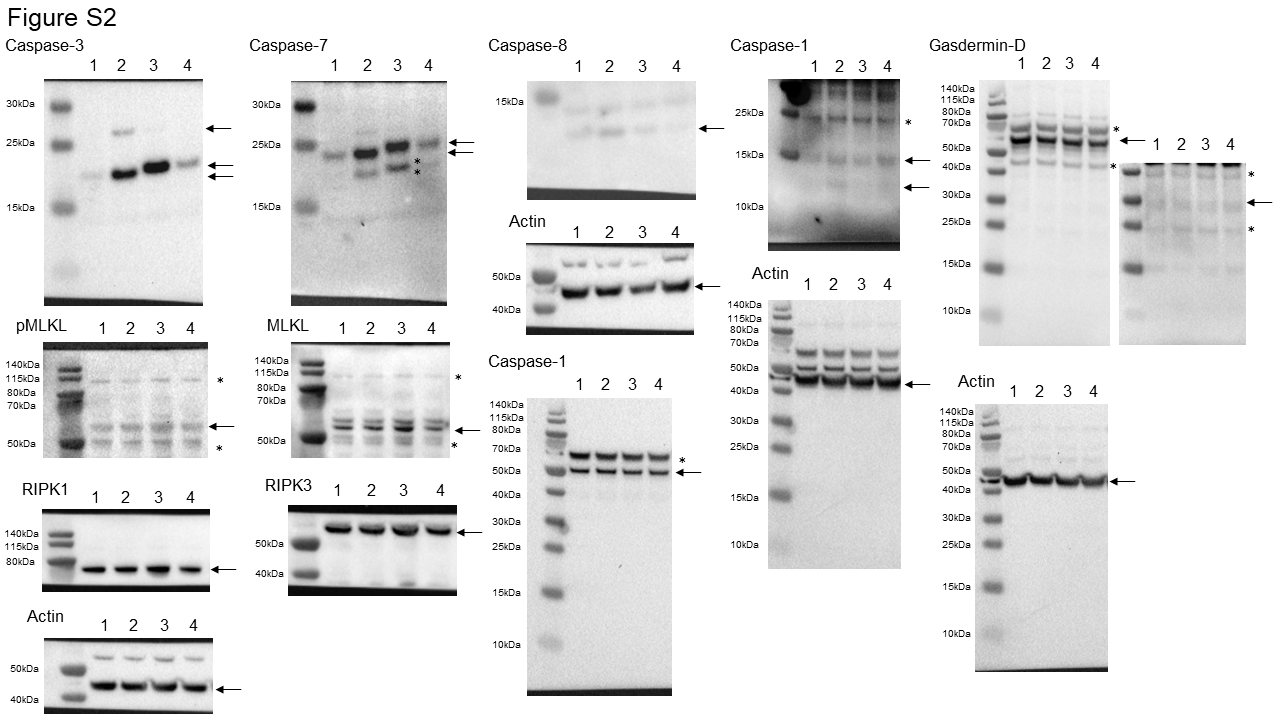

Supplement: Supplementary Figure 2 — Uncropped images of western blot experiments. Full-length western blot images for the detection of caspase-3, caspase-7, caspase-8, caspase-1, pMLKL, MLKL, RIPK1, RIPK3, GSDMD and corresponding β-actin as presented in Figure3 and FigureS4. The represented lanes 1 to 4 are: 1: control, 2: 7-ketocholesterol, 3: 7-ketocholesterol + Z-IETD-FMK, 4: Z-IETD-FMK. Non-specific bands are marked with an asterisk. [file Image2.tif]

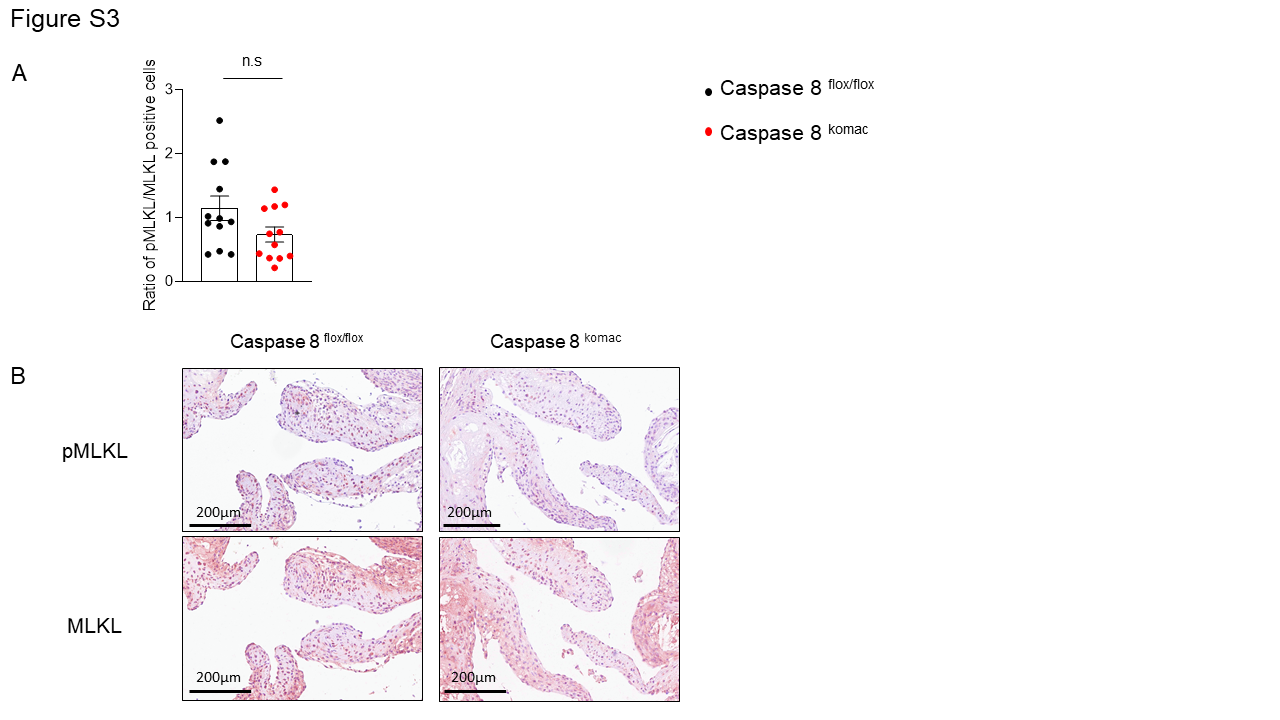

Supplement: Supplementary Figure 3 — Caspase-8 invalidation in myeloid cells impact the expression of pMLKL within atheroma plaque. (A). Ratio of pMLKL-positive cells to MLKL-positive cells in Casp8komac and Casp8flox/flox mice (n=12). (B). Representative images of pMLKL and MLKL staining in the valve of Casp8komac and Casp8flox/flox mice. MLKL and pMLKL positive cells are stained in red by the chromogen, while cell nuclei are counterstained in blue with hematoxylin. Statistical significance was analyzed with an unpaired-t-test. p values between experimental groups are depicted above the graphs. [file Image3.tif]

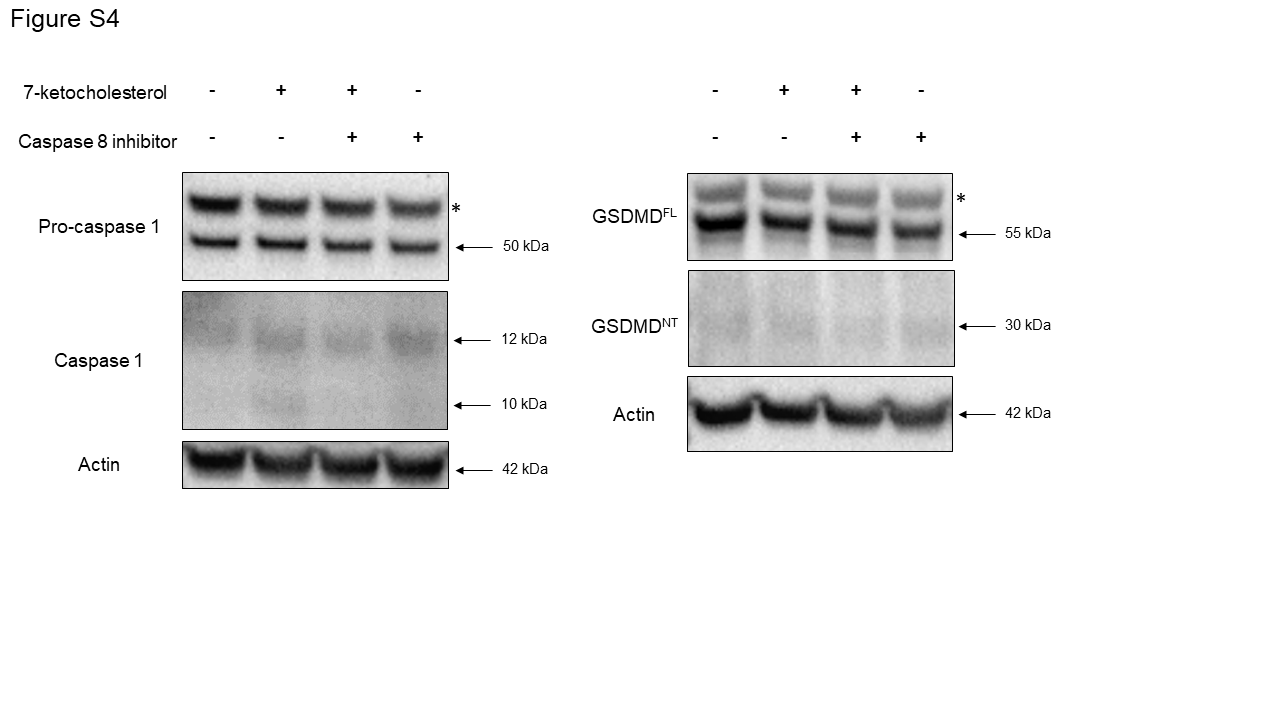

Supplement: Supplementary Figure 4 — Inhibition of caspase-8 in primary macrophages impact caspase-1 but not gasdermin-D cleavage. Representative western blot analysis of pro-caspase-1, cleaved fragment p12 and p10 and gasdermin-D, full length (FL) and N-term fragment (NT), in BMDMs from C57/BL6J mice exposed to 40µM 7-ketocholesterol, with or without Z-IETD-FMK, for 18 hours. Nonspecific bands are marked with an asterisk. β-actin was used for protein normalization. [file Image4.tif]
